# Improvidence, Precaution, and the Logical-Empirical Disconnect in UK Health Policy

**DOI:** 10.1007/s10728-022-00450-8

**Published:** 2022-12-26

**Authors:** Jordan A. Parsons

**Affiliations:** 1 Keele Law School, Keele University, Keele, UK; 2 Bristol Medical School, University of Bristol, Bristol, UK

**Keywords:** Abortion, Evidence-based policy, Health policy, Organ donation, Precautionary approach

## Abstract

The last decade has seen significant developments in UK health policy, with are largely claimed to be evidence based. However, such a characterisation ought, in many cases, to be questioned. Policies can be broadly understood as based primarily on either a logical or empirical case. In the absence of relevant empirical evidence, policymakers understandably appeal to logical cases. Once such evidence is available, however, it can inform policy and enable the logical case to be set aside. Such a linear policy process is not always the reality, and logical cases often continue to guide policy decisions in direct opposition to empirical evidence. In this paper, I discuss two recent examples of this disconnect between logical and empirical cases in UK health policy. The first—organ donation—illustrates an example of a significant policy change being made in opposition to the evidence. I refer to this as the improvidence approach. The second—abortion—provides an example of policymakers not making a change that has extensive supporting data. I refer to this using the more recognisable language of the precautionary approach. Ultimately, I argue that both the improvidence and precautionary approaches are examples of problematic public policy where policymakers provide no explicit justification for going against the evidence.

## Introduction

The last decade has seen significant developments in UK health policy. Beyond general movement in the direction of person-centred care—as typified by NHS England’s Long Term Plan [34]—more specific policy changes have taken place in, for example, the areas of organ donation [49] and abortion [52]. Such policies are often badged as evidence based in pursuing specified goals, yet the extent to which such a characterisation is appropriate can be legitimately questioned in many cases.

When justifying policy decisions, policymakers often appeal to what I will refer to as logical and empirical cases.^1^ This is not a stark distinction, as I will soon explain, but a matter of emphasis. Understandably, logical cases tend to come first—thought processes, not unlike Rawls’ idea of considered judgements [56], play out before research shows such judgements to be empirically supported (or not). Once research confirms or denies a logical case, it may be argued that the empirical case *should* then take precedence in pursuit of evidence-based policy, or, at the very least, be actively acknowledged as set aside for a particular reason. Such evidence may be qualitative or quantitative in nature and may be locally collected or based on comparative evidence from other jurisdictions. Frequently, however, logical cases appear to guide policy decisions in direct opposition to corresponding empirical cases. At this point, we might consider such cases *quasi*-logical; they maintain an air of logic and perhaps a valid argument, but the introduction of opposing empirical evidence renders them unsound. I will explore this distinction further shortly. I also note here that, inevitably, there will ordinarily be several logical cases concerning a policy issue—even expert opinions frequently differ as to what is to be expected when forecasting impact. As such, hereafter any reference to the logical case for a particular policy refers to that which is most dominant in the policy arena and which policymakers appear to endorse.

In this paper, I discuss two recent examples of this disconnect between logical and empirical cases in UK health policy. Whilst I discuss only two examples in this paper, the pertinent public policy point applies widely. The first example—organ donation—illustrates a significant policy change being made in direct opposition to the evidence (or, arguably, in the absence of evidence). I refer to this as the improvidence approach. The second—abortion—provides an example of policymakers *not* making a change that has significant backing from the medical community based on extensive data. I refer to this using the more recognisable language of the precautionary approach. In both cases, policymakers have gone against evidence that speaks to the policy goals they have themselves articulated. Ultimately, I argue that both the improvidence and precautionary approaches are examples of problematic public policy in the way they tend to arise, and that they are justified only where there is an explicit response to the evidence by policymakers.

## Logical-Empirical and Improvidence-Precaution

Before considering specific examples, it is prudent to clarify certain concepts that are central to this discussion. Specifically, what I mean by logical and empirical cases, and by improvidence and precautionary approaches.

As noted above, logical cases may exist independent of any empirical evidence. A logical case may contain empirical claims, but they are as yet unconfirmed. An empirical case, by contrast, is founded upon existing evidence. The key distinction, then, is evidential support. Nonetheless, there are other aspects to highlight.

First, logical and empirical cases may bleed into one another; they are not as cleanly distinct as they may first appear. That is because a logical case may include some empirical evidence and an empirical case will most likely include some logic. For example, a logical case may seek to draw a very loose comparison with something else for which there is existing evidence. What precludes this being deemed an empirical case is the proximity of that existing evidence—it is about a different matter which may well prove very different in practice. As such, logical cases may rest on empirical *assumptions*, the difference being that an empirical case is based on empirical *evidence*.

Each policy matter is also not limited to one logical case and one empirical case. Of logical cases in particular there may be many, as people may disagree in their predictions as to a policy’s outcome in the absence of evidence. For example, two individuals may disagree as to whether a certain phenomenon will arise as a result of a particular policy, providing two logical cases to contend with until some future point where an empirical case may arise to confirm or deny such predictions.

Further, importantly, both should be recognised as value laden. Whilst based on some manner of empirical evidence, an empirical case cannot rightly be viewed as somehow detached and objective in the way it has more traditionally be presented per what Bijker, Bal, and Hendriks refer to as the ‘standard view of science’ [4]. A more accurate understanding of empirical evidence recognises that ‘various social and cultural processes play important roles in the human work that goes on’ in research [4]. Thus, both logical and empirical cases include value judgements. This is not necessarily problematic but is at the very least something to be conscious of. After all, policy making is inherently value laden, most easily discernible where a governing party’s policies track the feeling of their voters against expert advice. Unavoidably, this also means that my interpretation of the cases I later discuss is subject to my own value judgements.

The purpose of this logical-empirical distinction in this paper, then, is primarily highlighting where pertinent empirical evidence has supported a policy case. By pertinent, I mean that which speaks to a recognised policy goal. For example, if there was a goal to reduce road traffic collisions, a logical case may suggest that speed bumps are a good choice, whereas a later empirical case may highlight that, in fact, speed bumps increase collisions.^2^

An empirical case having such supporting evidence does not make it inherently more “right” than a logical case. It should not *automatically* trump a logical case purely because there is supporting evidence. However, it having evidential support, I suggest, is reason for an empirical case to be given serious consideration. In the policy process itself there is inevitably a need to tease out where values are at play and whether such values ought to be endorsed. But an empirical case should be afforded an initial normative privilege such that a decision to go against it requires suitable justification, else the resulting policy represents either the improvidence or precautionary approach.

**Fig. 1:**
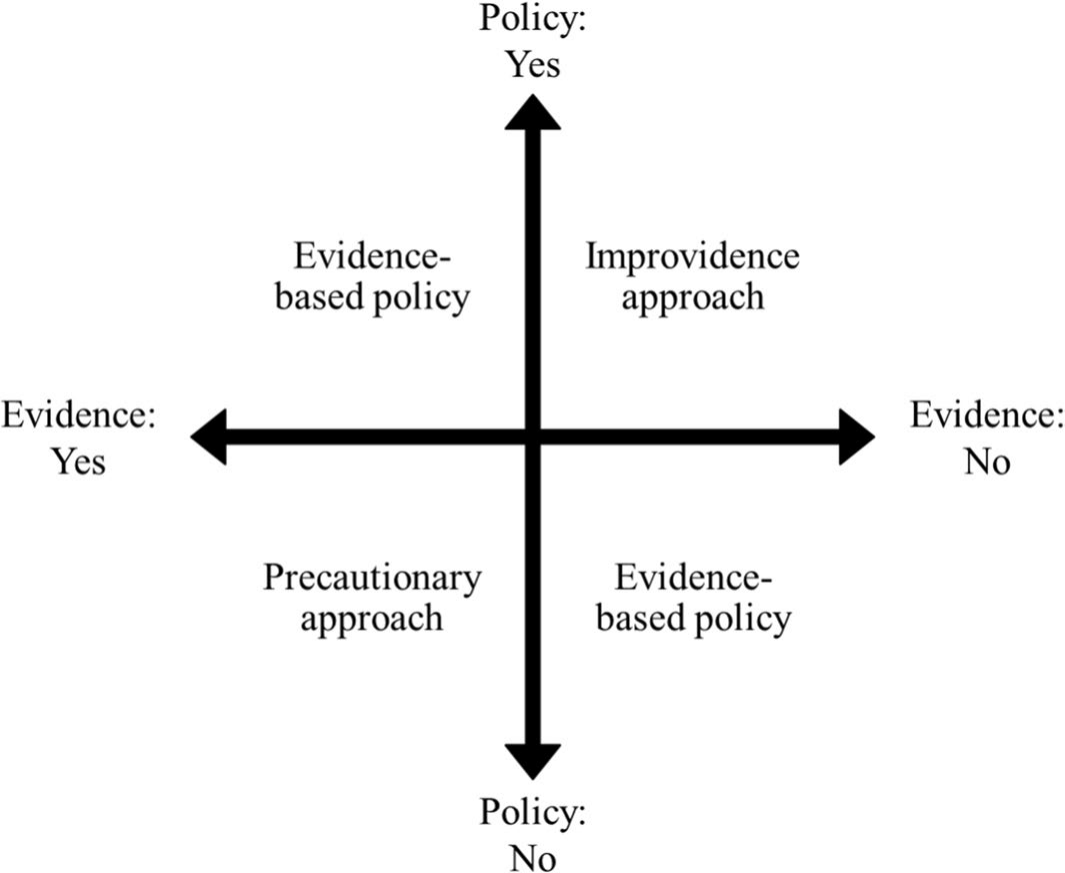
Policy approaches in relation

When I refer to the improvidence approach, I am describing instances where policymakers forge ahead with a given policy—i.e., departing from the status quo—where there is either no evidence or evidence that suggests such a policy will not achieve its goal. In some cases, available evidence may go as far as to suggest the policy will have the opposite effect to that sought. Pursuit of such policies is based on a logical case in which policymakers have placed significant confidence. There is a strong conviction that a given policy goal will be achieved despite there being either a lack of supporting evidence or the presence of contrary evidence. The improvidence approach is represented in the top right quadrant of Fig. 1; the evidence says no but the policy says yes.

The reverse of the improvidence approach is the precautionary approach. The terminology of “precautionary approach” has varied usage across disciplines, such that one cannot consider it to have a single, clear definition. Here, I use it to refer to decisions to maintain the status quo on a particular policy matter in the face of compelling evidence to suggest a change. The reasoning behind such failure to act is generally that of avoiding possible harms that the policy in question may cause. However, per the formulation of the precautionary approach deployed here, this is excessive caution as the compelling evidence necessarily addresses potential harms.

More specifically, the precautionary approach is to do so where the change suggested by such evidence is likely to bring about a policy outcome that policymakers have explicitly stated to be a goal. This additional qualification is important to exclude instances where policymakers knowingly go against evidence with some other goal in mind—most likely a political goal, pursuing a policy they believe will be popular with voters—regardless of the ultimate result. The precautionary approach is represented in the bottom left quadrant of Fig. 1; the evidence says yes but the policy says no.

Essentially, both the improvidence and precautionary approaches are examples of a misalignment between evidence and policy. Where there is a greater alignment, this can be considered evidence-based policy, as represented in both the top left and bottom right quadrants of Fig. 1.

## The Case of Organ Donation

First, to consider the policies on organ donation across the UK. More specifically, the implementation of systems of deemed consent for organ donation (I proceed with the terminology of “deemed consent”, though different terminology is used across jurisdictions).

Deemed consent in organ donation is a policy whereby people are taken to agree to donating their organs when they die in the absence of a recorded decision to the contrary. Such a system makes donation the default, shifting the onus to those who *do not* want to donate rather than requiring those who *do* want to donate (who are generally considered to be the majority) to make this known. Whereas there is disagreement as to finer points of the policy, it is generally considered defensible where there is widespread awareness of the policy and people can, without unreasonable difficulty, formally record their objection to donation [59]. Deemed consent systems are broadly divided into “soft” or “hard”, with the distinction resting on the role of the family at the time of the donation conversation—this will be revisited shortly.

The logical-empirical disconnect in this context is such that policy has been introduced in the face of mixed (or potentially even opposing) evidence. Where policymakers pursue deemed consent policies, the underlying policy goal is almost always stated to be an increase in the availability of organs for transplantation. Recognising the global shortage of transplantable organs, policymakers seek to save lives by addressing this,^3^ often turning to deemed consent. In the third reading of the Organ Donation (Deemed Consent) Bill, Dan Jarvis MP explained that ‘the bottom line is that it will save lives’ [12]. When asked, a year after its coming into force, whether the new system had increased organ availability, Minister for Care and Mental Health, Helen Whately MP, explained that it had [11], despite the data showing that rates were simply increasing in a return to pre-pandemic levels [35]. The intentions behind the legislation are very clear.

Even before its implementation throughout the UK, evidence to suggest deemed consent achieves what it is intended to—i.e., an increase in the availability of organs for transplantation—is limited, with “success stories” being, arguably, negligibly related to the change of organ donation consent policy [20, 63]. Nonetheless, the three nations comprising Great Britain have now all introduced deemed consent [27, 29, 43], with Northern Ireland due to soon follow [38, 45].

### The Logical Case

With deemed consent for organ donation, the hope is that it will reduce (or maybe even entirely overcome) the shortfall of transplantable organs that results in hundreds of deaths every year in the UK [36]. The logic—and empirical assumption—of deemed consent is that it will remedy the disparity between the proportion of the population that say they would be happy to donate and the proportion that join the organ donor register by making them default donors. In England, for example, the Government’s response to the public consultation on deemed consent highlights how 80% of people in England say that they are willing to donate their organs but only 37% had recorded this decision formally [15]. By placing the burden to act on those who do *not* want to donate, deemed consent is expected to overcome this disparity and increase the availability of organs for transplantation, thus saving more lives [5].

The particular model considered—and ultimately introduced—in Great Britain is so-called “soft opt-out” [46, 49] meaning it does not strictly enforce the deemed nature of consent in all circumstances. Soft deemed consent systems afford those close to the deceased (i.e., family and friends) some role in the final decision, generally (as in Great Britain) by allowing them to prevent donation proceeding if they can evidence that the deceased would have objected [64]. Further, professional guidance in both England and Scotland states that donation should not proceed if the family (or other appropriate persons) cannot be contacted, even if the conditions are such that it would technically be lawful [28, 61]. It is this safeguard that, per the logical case, prevents significant public distrust in the system as was the case in Brazil when a hard system was introduced (eventually resulting in its revocation) [9]. That being said, there are examples (albeit few) of hard systems of deemed consent lasting—notably Austria and Singapore. It is the distinction between soft and hard systems of deemed consent that can be considered the cause (or at least *a* cause) of the disconnect between the logical and empirical cases—this I will revisit shortly. Indeed, it may even be considered an internal disconnect whereby the logical case appears largely premised on hard deemed consent whilst the actual policy is soft deemed consent.

So, the logical case for deemed consent is that anyone who wants to donate becomes a donor, and only those who oppose donation are excluded. This is expected to increase the supply of transplantable organs and save lives. It is easy to see how such logic arose, as such an outcome certainly seems plausible. Indeed, with appropriate safeguards, it can also be considered ethically defensible. The logical case’s anticipated outcome, however, is not universally the experience where the policy has been implemented, and where data have been drawn on in support they have largely been framed through a very generous interpretation.

### The Empirical Case

The evidence concerning deemed consent for organ donation is, by and large, that it does not work. Or at least that it does not work *in isolation* [57]. Spain is a common point of reference when discussing deemed consent’s potential impact on transplantation rates. For years, Spain has been recognised as a leader in organ donation, and for good reason—it remains in first place both in terms of donors after cardiac death and donors after death by neurological criteria [21]. Given these rates and the fact that, since 1979, Spain has operated a system of deemed consent, the former is often assumed to be a direct result of the latter. However, the reality is quite different, such that making this connection is conflating correlation and causation [63].

Spain invested significantly in improving its system of organ donation in 1989, including a central role for transplant coordinators at all organ procurement hospitals. With resources dedicated to finding potential donors and early approaching of families, the Spanish system enables an environment in which donation rates can organically increase. The role of the legislation itself, then, can be brought into question—at most, it can be thought of as one of several contributors to the increase. Even some of those who are supportive of legislative change acknowledge that it is insufficient, and that appropriate financial investment and organisational changes are necessary to achieve results [19]. What appears to be the case is that, in Spain, the procedural changes in organ procurement are responsible for the impressive statistics, suggesting that legislative change itself is unnecessary [20]. Such legislation may instead be considered a principled addition to indicate a positive attitude to donation.

I will come to detail the legislative changes in Great Britain in the next section, but it is worth briefly highlighting the empirical reality following the introduction of deemed consent in Wales. Analyses thus far have shown that the legislation has not improved donation rates in Wales [40, 46]. Whilst it may still be considered early days—the Welsh system having come into force in December 2015—one would have expected at least a modest improvement by now if the logical case outlined above were to be accurate. In England, too, as noted above, what is being viewed as an increase is more accurately taken as rates returning to where they were before COVID-19 [35].^4^

### The Policy

Deemed consent is now in operation across Great Britain. Wales introduced the policy first [29], followed some years later by England [43] and, most recently, Scotland [27]. Whereas Northern Ireland does not currently have such a consent system for organ donation, it is soon to be implemented, which will result in the whole of the UK being on largely similar footing—the Organ and Tissue Donation (Deemed Consent) Act (Northern Ireland) 2022 is due to come into force in spring 2023 [38, 45].

The introduction of deemed consent across Great Britain was seemingly based on the logical case outlined above, despite the empirical case having highlighted its inaccuracies—notably, that such a change in legislation does not necessarily overcome the shortage of transplantable organs. This is apparent in the way many policymakers have discussed the change, speaking in broad terms about saving many lives without acknowledging the mixed evidence. It should also be noted that these changes came about even though the Organ Donation Taskforce—an independent group tasked by the 2007 government to consider the implementation of an opt out system—advised against the policy change after reviewing it in depth [44].

Wales introduced its system long before England and Scotland, so whilst strong evidence still existed, it could at least be attributed to some level of optimism that Wales was somehow an exception (cultural differences play a part in the success of organ donation programmes, so examples from abroad are not perfect predictions). However, the decision in England and Scotland came after the realisation that the impact of the system in Wales was something of a let-down. Despite the realistic expectation that donation rates would not increase because of the legislative change, Matt Hancock, on the day of the new system’s introduction in England, said that the change was a ‘milestone for organ donation’ which would mean that ‘hundreds more lives could be transformed each year’ [37]. With its neighbouring nation, with which it shares much of its legislation, seeing no results, England still pursued the policy whilst vocally continuing to draw on the logical case. Perhaps more problematically, England proceeded to introduce deemed consent during the height of the COVID-19 pandemic, when the conditions for the ethical justification of such a system could not be guaranteed to be met [50]—but that is an altogether different discussion.

There has now been discussion of preliminary data since the implementation of the policy, with policymakers framing it positively to suggest that the policy has been a success. As noted above, Helen Whately MP has, in Parliament, spoken of how 29% of donations in England since the Act coming into force have been based on deemed consent [11]. Her comments are, however, an overly optimistic interpretation of the data. Under the previous opt in system, families were still able to consent to donation where the deceased was not a registered donor. There is no indication that this proportion of donors now deemed to consent would not have become donors under the previous system as a result of family consent, and we must assume that at least some of them would have been. Further, it is possible that some of those deemed to have consented were not on the organ donation register *because of* the new system; they may have been willing donors who did not consider it necessary to make this explicit because they were aware their consent would be deemed anyway. Essentially, the data do not answer many of these more specific questions that are essential to a fair interpretation of the policy’s impact, and policymakers using them to claim success are seemingly attempting to present the logical case as an empirical case to garner further credibility.

Deemed consent in Great Britain (and, soon, the whole of the UK) can, then, be considered an example of the *active* logical case being favoured over the *passive* empirical case. Despite data suggesting the policy will not have the desired effect, policymakers forged ahead with its implementation based on its theoretically intended effect. This is an example of the improvidence approach, represented in the top right quadrant of Fig. 1; the data say no but the policymakers say yes.

## The Case of Abortion

A disconnect is similarly apparent in UK abortion policy, though in a slightly different—and, I suggest, more problematic—way. In this context, the implementation of policies with a wealth of historic and emerging data have been significantly ignored (or, in a sense, delayed). Whereas deemed consent was introduced in the absence of concrete supporting evidence, it can at least be reduced to “taking a punt on the policy” and, arguably, is not (significantly) actively harmful. Delaying (or entirely preventing) the implementation of well-evidenced policies relating to safe access to healthcare without adequate justification, however, actively negatively affects some proportion of the population—oftentimes denying access to (potentially lifesaving) care. As such, the case of abortion discussed here is far more concerning.

Here, I discuss the approval of home use of both mifepristone and misoprostol for the purposes of telemedical early medical abortion (TEMA). Early medical abortion refers to the termination of pregnancy using the abovementioned medications—ideally taken between 24 and 48 hours apart—in the first 10 weeks of pregnancy (it is not a strict definition and is sometimes used to include treatment up to 12 weeks’ gestation). The first drug (mifepristone) causes the lining of the uterus to breakdown, thereby preventing the pregnancy from continuing. The second drug (misoprostol) then triggers uterine contractions, causing the expulsion of the products of conception. Early medical abortion (though not necessarily at home and/or utilising telemedicine) is a common method of abortion in Great Britain, accounting for 87% of terminations in England and Wales and 99% in Scotland [41, 54].^5^

There has long been strong evidence supporting the practice of at-home early medical abortion and TEMA, drawn from several countries and care pathways. It focuses on the safety, effectiveness, and acceptability of treatment, with comparable (or better) outcomes to more traditional, in-person care—which I will soon detail.

Throughout various debates about abortion care in the period of change concerned, policymakers have stressed that their focus is on the safety of those accessing care. They have, however, consistently failed to introduce these changes when the suggestion has arisen, instead indicating, in the face of a strong evidence base, that we cannot ensure the safety of patients with these new care pathways—where they have eventually been introduced, it has been as a result of significant pressure, either with neighbouring nations having done so or with the COVID-19 pandemic making traditional care pathways unworkable [51]. There is no denying abortion is a highly contentious policy matter which is often polarising. Nonetheless, that policymakers note the importance of patient safety yet drag their feet on a policy that has been consistently shown to improve treatment outcomes highlights a highly precautionary approach to such policy—it is denying and ignoring evidence that points to a goal they claim to be in pursuit of.

### The Logical Case

There has been significant opposition to the introduction of TEMA. Whilst some concerns are common to telemedicine in any context—questions about cyber security and damage to the doctor-patient relationship [48]—others have been raised that are specific to TEMA. Such concerns have been used to weave a narrative against changes to enable these services, which can be taken to form the dominant logical case in this context.

In large part, the dominant narrative has been pushed by pro-life organisations Christian Concern and the Society for the Protection of Unborn Children (SPUC). In a briefing document published by SPUC, the claim that it is ‘completely unnecessary for women to attend a clinic to take a pill’ is deemed wrong on the basis that clinic attendance is about more than just administering the drugs [66]. The document notes that clinic attendance is important to date the pregnancy (by ultrasound scanning) and ‘help to ensure that [the patient] is not being coerced’ [66]. Similar views were seen in Parliament in debates over the Coronavirus Bill (later Coronavirus Act 2020). In the House of Lords, when faced with willing from some Lords to incorporate amendments to ensure access to abortion care, Lord Bethell responded:

we do not agree that women should be able to take both treatments for medical abortion at home. We believe that it is an essential safeguard that a woman attends a clinic, to ensure that she has an opportunity to be seen alone and to ensure that there are no issues. […] The bottom line is that, if there is an abusive relationship and no legal requirement for a doctor’s involvement, it is far more likely that a vulnerable woman could be pressured into have an abortion by an abusive partner [10].

Clearly, then, the dominant logical case guiding the policy narrative concerning TEMA is opposed to the change based on safeguarding concerns and the accuracy of gestational age estimates in the absence of in-person care. Such sentiment is so strong that, when TEMA was temporarily introduced, Christian Concern launched a legal challenge that reached the Court of Appeal [55]. Whilst this challenge was unsuccessful, it demonstrates the significant backing of the logical case.

At first, this logical case makes sense. One would anticipate that accessing care remotely and undergoing the treatment at home could raise safety concerns, and it is appropriate that policymakers are cautious of inflicting harm through policies where such a risk is present. To not be an “early adopter” in terms of such progressive abortion care pathways is, then, justifiable. However, on this policy there was a long-term failure to adopt it, favouring this logical case even when it was found not to align with empirical evidence.

### The Empirical Case

Here, the empirical case is far stronger than with organ donation. Evidence regarding TEMA is, and long has been, strongly supportive of the policy. The method itself (meaning early medical abortion, distinct from the specifics of the care pathway) is strongly recommended by the World Health Organization up to nine weeks’ gestation—the Organization also recommends the method up to 12 weeks’ gestation, though as a weaker recommendation [70]. A 2019 systematic review of TEMA found it to be safe, effective, and acceptable to patients, collating data from 13 studies dating back as far as 2008 [17]. Many studies have assessed the Women on Web service, established in 2005 to provide TEMA where abortion is illegal or difficult to access [69]. These studies have overwhelmingly found high levels of acceptability and patient satisfaction across jurisdictions (including Brazil, Hungary, and Ireland) [2, 24, 31]. More recent data from Great Britain—since the establishment of TEMA services—corroborate these findings [3].

Concerns raised about TEMA services in relation to confirmation of gestational age and safeguarding are similarly unfounded. Studies demonstrate that patients can estimate gestational age based on menstrual history within an acceptable^6^ margin of error—with accuracy increasing as does the gestational limit on accessing care [6]. Further, data from the early days of TEMA in England and Wales show that care is being accessed earlier in pregnancy [14], thereby rebutting the suggestion that abortions will be carried out at home well into the second trimester. As for safeguarding, there is no reason why it cannot be satisfactorily performed through remote consultation [53, 58]. The British Pregnancy Advisory Service - one of the UK's largest abortion care providers - has seen an increase in enhanced safeguarding risk assessments since establishing its TEMA service [7], suggesting a somewhat cautious approach to care provision that may *better* discover cases of concern [53, 58]. Preliminary—though as yet unpublished—results of a recent study also indicate that safeguarding practices incorporated into new telemedical care pathways are proving effective, in some cases enabling professionals to identify patients with safeguarding concerns that in-person care may not have.^7^

In sum, the evidence overwhelmingly demonstrates that TEMA is safe, effective, and acceptable to patients. Further, more specific concerns around dating pregnancies and maintaining effective safeguarding are empirically unfounded. The empirical case is, then, heavily in favour of the availability of TEMA services. The emergence of such data has undermined the earlier logical case.

### The Policy

Policymakers have historically been slow to progress abortion policy in the UK—and, incidentally, this is largely true across the world. That MPs are (at least formally) afforded a free vote (or conscience vote, meaning MPs are not whipped to follow the party line) on any change to abortion law [26] is testament to how morally charged this area of policy is. The move to permit home use of misoprostol (the first drug used) only began with Scotland in 2017 [47], with Northern Ireland only having followed suit in 2020 following interference by Westminster [52, 67]. This was long after such practice was commonplace in many jurisdictions around the world.

More recently, temporary approval orders were issued across Great Britain (though *not* in Northern Ireland) to permit TEMA [13, 39, 52, 60]. The policy being discussed is, then, at least in Great Britain, in place—the empirical appears to have trumped the logical. However, these approvals were the result of intense pressure in the context of the COVID-19 pandemic [42]. Further, they were initially introduced only on a temporary basis, with England and Wales having only recently removed sunset clauses to make them permanent (the Scottish approval remains temporary in principle, with an indicated intention to review the situation after the COVID-19 pandemic). That these approvals were not initially issued as permanent changes or introduced prior to the pandemic shows that policymakers continued to cling to the logical case despite the wealth of evidence in favour of TEMA, and it remains feasible that these regulations will be revisited in the not-too-distant future. One may even question whether these changes would have been implemented at all by now if it had not been for the pandemic. Whereas the COVID-19 pandemic can be considered to have added weight to the empirical case, it was sufficient even beforehand. In Northern Ireland, the dominant logical case does again appear to be prevailing as there have be no significant indications that the approval of TEMA—which would bring the nation in line with the rest of the UK—is forthcoming.

The ability of a policy based on a logical case to last in the face of opposing empirical evidence highlights the real strength such cases can have in contested areas of policy. That is not to say that a policy should be introduced purely because of empirical backing, but in this case the evidence pointed to the goal that policymakers claimed to be pursuing. As such, this example of TEMA’s slow and unsteady introduction is a prime example of the precautionary approach, represented in the bottom left quadrant of Fig. 1; the data say yes but the policymakers say no.

## Problematic Public Policy?

It appears that UK health policy sometimes entails some level of disconnect between the logical expectations of a policy and the empirical reality. This is, in some ways, an example of problematic policy, in particular because some instances can cause real harm to people. It is yet more problematic where policymakers vocalise the credentials of their policies as evidence based before praising them post-implementation as successes even where the data suggest otherwise. It must be remembered that an evidence-based policy is only as good as the “evidence” it is based on. This has become something of a caricature of the response to the COVID-19 pandemic in the UK which, incidentally, might be considered rather more arbitrary than even the pursuit of a disproven logical case.

In the two examples discussed, the logical-empirical disconnect is illustrated in different forms. With organ donation, the introduction of deemed consent in the face of, at best, mixed evidence is an example of what I term the improvidence approach. The delay in implementing/refusal to implement TEMA despite extensive and ever-growing evidence in support of the policy exemplifies the precautionary approach. Both can be problematic, but for different reasons.

Following the improvidence approach can be problematic because it has the potential to damage public confidence in policymakers, as well as wasting resources. If a policy is introduced with great fanfare—as was the case with deemed consent in Wales (though perhaps less so in England and Scotland due to the COVID-19 pandemic drawing media attention)—but ends up failing to deliver on expectations, the public may begin to question the decisions of policymakers [8]. One may consider this acceptable in the game that is politics, as policymakers can decide for themselves to take the necessary steps to understand the likely success of a policy before implementation. But beyond the politics, individuals who stand to benefit from a policy according to the logical case may be more directly harmed. Take, for example, the many people on the organ transplant waiting list, for whom the move to deemed consent was touted as a ray of hope. For these patients—and, indeed, their loved ones—the realisation that they are not markedly more likely to receive a transplant because of the policy change will likely prove devastating if they have been led, by politicians, to believe they would be.

Further, introducing policies for the purpose of achieving a goal that evidence suggests will not be achieved is a waste of resources. This might be considered poor practice in any setting, but, in the context of a publicly funded healthcare system with finite resources (such as the NHS), the costly implementation of ill-conceived policy is especially troubling because it takes away from important services. That this happens is perhaps an unfortunate symptom of short terms of office. If a government can garner a public relations boost in the short-term from the introduction of a particular policy—such as deemed consent for organ donation—then it may be less likely to consider the financial implications of that policy failing after significant public funds have been spent on it. After all, a new government may take the fall down the line.

The precautionary approach is a matter of, arguably, *greater* concern. With the precautionary approach, policymakers are actively choosing *not* to implement a policy that has been demonstrated to achieve the goal they have set; rather than making decisions based on limited or conflicting evidence, a substantial body of evidence is being ignored in favour of (disproved) theories about impact. It is the weight of evidence in relation to the stated policy goal that is crucial here, as the precautionary approach requires that available data be suitably extensive, thereby pushing it beyond the realms of justifiable caution. Where the implementation of the policy stands to benefit a particular demographic (as is generally the intention with all health policy) without simultaneously depriving another, a failure to implement it is causing harm. This was very much the case with TEMA in Great Britain (and remains the case in Northern Ireland); extensive evidence has long demonstrated TEMA to be safe, effective, and acceptable to patients, such that collective medical opinion is that it is appropriate care, yet it took a pandemic for the policy to be (initially temporarily) implemented in Great Britain and it still has not been implemented in Northern Ireland.

Understandably, policymakers want to be confident that a policy does not end up harming people. This is certainly something of a moral imperative here, and a light touch precautionary approach—whereby a policy discussion is delayed due to *severely* limited evidence—is not *necessarily* cause for concern. Indeed, this ought not to be deemed an example of the precautionary approach at all, but justifiable caution. However, where the evidence quite strongly suggests that no such harms will materialise (as is the case with TEMA), a failure to act constitutes the causing of harm. It is in that sense that Northern Ireland is harming pregnant people who are seeking abortions, and that Great Britain was. This is a strong example of the precautionary approach that is unjustifiable both in an ethical sense, but also in a political sense where policymakers want to be viewed as following the evidence (which they so frequently claim). It is a particularly zealous extension of the maxim “better safe than sorry” that, in effect, turns out to be better *un*safe than sorry. The particular example of TEMA may also be considered *yet more* concerning for the fact that its introduction required (or requires, in Northern Ireland) the approval of only the relevant health minister—the full parliamentary process is not required because of particular provisions included in primary legislation for this exact purpose [1, 67].

In addition to recognising that such approaches to policy are problematic, one must consider *why* they are adopted. When it comes to the precautionary approach, the influence of moral conservatism (often of an explicitly religious nature) is sometimes apparent. That two influential groups in the abortion debate—SPUC and Christian Concern—are explicitly religious demonstrates this. Moral conservatism (and religion) in public policy is certainly a complicated issue [18]. However, it might be considered problematic when such influence pervades the dominant logical case that is pursued contra empirical evidence. This is not the place for a detailed exploration of the role of moral conservatism in abortion politics, but it cannot be ignored that part of the reason for the excessive precautionary approach with abortion policy in the UK (particularly Northern Ireland) is problematically attributable to such influence. Of course, other considerations in policymaking are always present—and justifiably so. But where they stand in opposition to empirical evidence there ought at least to be acknowledgement of this reality; if favouring, for example, a particular moral position in a policy decision, this should be made clear, rather than the invocation of a reliance on poorly interpreted or non-existent data. This is, above all, a matter of transparency.

With the improvidence approach, moral conservatism is perhaps less overtly pertinent. Whilst the deemed consent debate considered religious groups—the public consultation in England prior to the change in policy asked about the impact on people from some religious groups, and 33% of respondents stated that they felt the impact would be negative [15]—strong opposition to the change from such groups did not arise. It might be, then, that, in the absence of significant opposition of a morally conservative nature, policymakers were happier pursuing the deemed consent policy. This is, however, conjecture, and I do not wish to conclude the precise role of moral conservatism’s influence in these policy decisions here. Further, even if moral conservatism can be said to influence the pursuit of the precautionary and/or improvidence approaches, it certainly cannot be said *always* to influence; it may be relevant to the particular issues discussed in this paper, but some policies simply will not raise significant moral concerns.

Whilst I have undoubtedly presented myself as an advocate of evidence-based policy, I would stress that I do not propose that policymakers ought to be doggedly implementing all policies with a compelling empirical case. Empirical support for policies is important, but a strong empirical case can be made for many abhorrent policies that one would hope policymakers would disregard on other bases. Equally, ‘technological expertise cannot be relied upon to discover the characteristic risks and the social implications of new technologies’ [33], which are arguably as important as ensuring a policy achieves what it seeks to. Other considerations are important.

The idea of evidence-based policy has a long history, with links to early modern ideas around ‘effective statecraft and efficient governance’ [25]. But, as explained by Shaxson, ‘[e]vidence is a necessary, but not a sufficient, condition for any decision-making process’ [65]. Under the first Blair Government, the Cabinet Office stated that ‘policy decisions should be based on sound evidence’ but acknowledged that sources of relevant information might include things such as stakeholder consultation [68].^8^ In a democracy, what people think of a policy also holds value, and stakeholder perspectives should not be set aside in cold pursuit of empirical cases; policy decisions result from ‘an interplay of facts, norms and preferred courses of action’ [25].

My concern is not that empirical cases are not always being taken up. It is more that the way empirical cases are seemingly viewed in UK health policy is overly dismissive—that is where they are viewed at all by policymakers. There does not appear to be genuine consideration of the evidence, making a decision to go against it questionable. Thus, Hunter’s point about the uptake of public health research in policy being ‘variable in the extreme’ [30] ought not *necessarily* be a matter of concern, as we should be more focused on the process.

Alternatively, I suggest there is a need for something akin to Rawls’ reflective equilibrium, whereby coherence is sought by at least justifying the setting aside of a given (empirically informed) position. What is observed through the improvidence and precautionary approaches, particularly in the examples I have explored, is a lack of coherence. Policymakers declare a given policy goal, but then pursue a course of (in)action that evidence suggests will not bring about that goal—or, worse, will undermine that goal. Going against an empirical case is certainly justifiable, but, I suggest, ought to require explicit justification; why is the pursuit of evidence-based policy not appropriate in that circumstance?

Such a critical approach to the policy process is important to the defensibility of resulting decisions. As in reflective equilibrium, one should give due consideration to conflicting positions in arriving at a final, coherent decision. The nature of policy is that consensus is almost always impossible—people will continue to fundamentally disagree about the importance of different values. However, we can hopefully agree on the importance of coherent positions and giving opposing views a fair hearing. Here I would agree with the position of Lindblom that the policy process can be evaluated only by standards of ‘fairness, acceptability, openness to reconsideration and responsiveness to a variety of interests’ [32]. However, I suggest this be caveated by affording empirical cases an initial privilege in considerations. Whilst it is important that both logical and empirical cases are explored—i.e., we should still take seriously the views that lack evidential support as they may be rooted in values we consider worthy of endorsement—it does at least seem reasonable that empirically founded positions be afforded a higher burden of proof for dismissal. This initial privilege enables evidence-informed rather than evidence-based policy, which may be ‘the best that can be hoped for’ [30].

I recognise that in some policy matters things are far more ambiguous and there is too little evidence to piece together a robust empirical case. In such scenarios, policymakers may hold off until the evidence base grows—a more justified caution. However, with this approach, it must be stressed that waiting for further evidence of the policy *in practice* necessarily requires that it be implemented somewhere. As such, a country that wants to be perceived as trailblazing in the realm of evidence-based policy may consider tentatively introducing such a policy to contribute to the body of evidence such that a more definitive decision can be made. This will enable other countries that are perhaps more embracing of a precautionary approach to benefit from a strengthened evidence base in either replicating the policy or not—in essence, policy transfer [16]. Equally, however, the logical case sometimes must be sufficient. Particularly when it comes to new policy ideas based on new interventions, there may be no empirical data available to inform decisions. In this situation, the introduction of a policy in the absence of compelling evidence can be appropriate, but it is important for the dominant logical case to be critically engaged with to develop the most realistic prediction of the policy’s impact.

## Conclusion

There is an extent to which I am asking the reader to agree with my assessment of the two policies discussed, which, despite the evidence provided, some inevitably will not. However, even if one does not consider the examples deployed fit for purpose, the point being made remains true. There are various other health policies that could be substituted in seamlessly to demonstrate these phenomena. Indeed, whilst my focus has been on health policy, it would come as no surprise if parallels could be drawn with other areas of public policy—though I will not comment on any such resemblances for lack of expertise.

Ultimately, my suggestion here is that this observable disconnect between the logical and empirical cases surrounding matters in health policy, alongside the somewhat arbitrary application of both the improvidence and precautionary approaches, should be viewed as problematic for the fact that policymakers are not showing their workings, so to speak. For policy to be considered evidence informed—which is a reasonable ideal to aim for—empirical evidence cannot be quietly brushed aside in favour of a minority’s (oftentimes morally conservative) opposition. Equally, brazenly adopting policies that sound good in theory but have, where already introduced, not met such high expectations ought to be questioned. The latter, however, ought to be thought of as *less* problematic—but problematic nonetheless. Whilst there may be justifiable reasons for going against the evidence in relation to a given policy goal, the decided upon policy ought only to be considered justified if it explicitly responds to that evidence. If another reason is prioritised over an empirical case it should at least be clear that this has happened and why.

## Footnotes

1 The two are not mutually exclusive, as I will come to discuss shortly.

2 I should stress at this point that I am not here providing an appraisal of speed bumps—this is purely for explanatory purposes.

3 Or, if one is feeling cynical, they seek the publicity benefits associated with trying to save lives.

4 Though it should be noted that any interpretation of organ transplantation data from 2020 to now is muddied by the pandemic impact, such that it is hard to differentiate precisely where an increase is a return to normal and where it is a result of the new policy.

5 Whilst with deemed consent I look to the whole of the UK, my primary focus here is on Great Britain. Due to a history of limited abortion access in Northern Ireland, the practice of early medical abortion does not have the same history of usage there.

6 Though I acknowledge that acceptability here is somewhat subjective.

7 The SAPHE Study is a qualitative study of safeguarding practices in new telemedical care pathways for early medical abortion at both BPAS and MSI Reproductive Choices. Its preliminary results have been presented at select events but are pending publication.

8 New Labour’s approach to evidence-based policy has, however, come under criticism. Sennett has made the accusation of policy consumption, whereby policymakers ‘behaved like consumers of policy, abandoning them as though they have no value once they exist’ [62].
